# Altered Apoptosis in Endometriosis Compared with Ovarian Carcinoma

**DOI:** 10.3390/medicina61111983

**Published:** 2025-11-05

**Authors:** Ozgur Ozdemir, Atila Yildirim, Yavuz Tekelioglu, Safak Ersoz, Suleyman Guven

**Affiliations:** 1 Department of Obstetrics and Gynecology, 7M General Hospital, Trabzon 61300, Turkey; 2 Department of Medical Oncology, School of Medicine, Karadeniz Technical University, Trabzon 61080, Turkey; atilayildirim@ktu.edu.tr; 3 Department of Histology and Embryology, School of Medicine, Karadeniz Technical University, Trabzon 61080, Turkey; ytekeli@ktu.edu.tr; 4 Department of Pathology, School of Medicine, Karadeniz Technical University, Trabzon 61080, Turkey; sersoz@ktu.edu.tr; 5 Department of Obstetrics and Gynecology, School of Medicine, Karadeniz Technical University, Trabzon 61080, Turkey; sguven@ktu.edu.tr

**Keywords:** annexin V, endometriosis, flow cytometry analysis, ovarian cancer

## Abstract

*Background and Objectives*: Endometriosis has been shown to be associated with an increased cancer risk, and apoptosis may be important in the pathophysiology of endometriosis. To date, it remains unclear whether the tissue cell surface apoptosis marker (annexin V) is an important parameter in terms of cancer and endometriosis. This retrospective study aimed to compare endometriosis cases and ovarian cancer cases in terms of apoptosis and cell proliferation markers’ levels. *Materials and Methods*: In total, 65 (30 ovarian endometrioma, 35 ovarian carcinoma) paraffin blocks were taken for flow cytometric analysis. The flow cytometry analysis markers and annexin V staining levels were compared. *Results*: The G2/M stage cell ratio, S-phase fraction, proliferative index, aneuploidy cell ratio, and annexin V apoptotic index ratio were found to be statistically significantly lower in the endometrioma group compared to the carcinoma group. However, the G0/G1 phase cell ratio was found to be higher in the endometrioma group. According to the correlation analysis results, annexin V expression level showed a positive correlation with the G2/M cell ratio and S-phase fraction, while it showed a negative correlation with the G0/G1 level. In addition, as the apoptotic index increased, the cell aneuploidy rate also increased, which was statistically significant. When the apoptotic index was used to distinguish between endometrioma and ovarian cancer (cutoff value 16.05%), the sensitivity was found to be 94.3%, and the specificity was found to be 80%, which was statistically significant for cases below the cutoff value to be accepted as endometrioma. *Conclusions*: Apoptosis was reduced in endometriosis cases. The cell DNA activity was altered in endometriosis cases, as in cancer cases. Flow cytometric analysis can be used in the diagnosis of endometriosis even in paraffin-embedded tissues. The flow cytometric annexin V analysis provided results in an average of 30 min, making it a promising and highly sensitive differential diagnostic marker to distinguish between endometriosis and ovarian cancer.

## 1. Introduction

Endometriosis is a disease in which the endometrial gland and stroma settle outside the uterine cavity. They respond to the hormones of the reproductive cycle just as the endometrium tissue inside the uterine cavity does; they move through thickening, thinning, and shedding stages, causing dysfunction in the tissue and where they settle. This causes symptoms of dyspareunia, dysmenorrhea, chronic pain, and infertility due to inflammation, adhesion, and mass effects. Endometriosis, which is frequently located in the ovary, is seen in approximately 10% of women of reproductive age. This disease is detected in approximately 50% of cases evaluated for infertility [1]. It continues throughout the reproductive age and has no definitive treatment. The treatment options are medical and surgical treatment approaches that aim to relieve symptoms and eliminate the organ dysfunctions causing the symptoms [2].

Insulin-like growth factor-1 (IGF-1), insulin-like growth factor binding protein-3 (IGFBP-3), leptin, the HOMA (hemostasis model assessment) score, fasting glucose, and calcium-125 can be used to differentiate between ovarian tumors and benign ovarian lesions. However, only low concentrations of IGF-1 and IGFBP-3 have been reported to be effective in distinguishing benign from malignant lesions [3].

Endometriosis can also affect the colon, the nearest organ. The characteristics of endometrioma, the patient’s history, and the surgical findings are important for staging colorectal endometriosis. Similarly, there is a close relationship between this staging and potential complications requiring treatment [4]. Therefore, because endometriosis is a widespread disease, causing morbidity, it is a focus of research.

Endometriosis treatment is also a focus of research. In addition to the well-known GnRH (Gonadotrophin-releasing hormone) agonist leuprolide acetate, many new antiangiogenic molecules have been introduced. Aflibercept, a new molecule with a similar molecular structure to VEGF, reduces and prevents choroidal neovascularization and tumor growth. It has also been reported to reduce endometriosis implant volume in experimental models of endometriosis and was shown to be more effective than leuprolide acetate [5].

Endometriosis has been reported to increase the risk of ovarian cancer 4.2 times [6]. However, the mechanism that increases this risk remains unknown. Oncogenic pathway activation and tumor suppressor gene inactivation have been reported as possible mechanisms [7].

Apoptosis is a cellular protective mechanism that involves the programmed elimination of unwanted and uncontrolled cells without causing any inflammation or immune response. Eutopic endometrial tissues have been reported to be resistant to apoptosis [8]. In our previous study, we reported that the apoptotic index in ovarian serous cystadenoma was lower than in serous cancer, but the cell ratios in the G2/M phase were similar in terms of flow cytometric analysis; therefore, serous cyst adenoma could be a precursor lesion of serous ovarian cancer [9]. Apoptosis may also be important in the development of cancer on the basis of endometriosis. In this context, it remains unclear whether tissue annexin V expression, an important marker for apoptosis, is a critical parameter in terms of cancer and endometriosis.

Apoptosis is programmed cell death, similar to necrosis. However, in necrosis, an inflammatory tissue response occurs along with distinct morphological changes. In contrast, apoptosis eliminates unwanted cells without triggering an immune or inflammatory responses. Therefore, it is a controlled process beneficial to tissue. In a disease or pathologic condition, the elimination of harmful, affected, and damaged cells or those from their functional purpose is critical [8]. In endometriosis, endometrial cells, which fulfill the basic menstrual regulation function in the endometrial cavity, settle in the ovary, causing disease, forming adhesions around the ovary, generating inflammation, contributing to organ function limitations, and causing pelvic pain, infertility, and even cancer development. The process of apoptosis for the controlled elimination of these cells is also crucial in endometriosis. This study provides current results on this topic.

The presence of apoptosis in endometriosis-associated cells can be demonstrated by two fundamentally different methods. The first is morphological assessment, which involves the detection of apoptotic bodies in apoptotic cells. The second involves biochemical changes involving alterations at the genetic level. Examples include the activation of cell death regulators, such as bcl-2 and caspases, leading to an increase in certain death proteins, as well as the activation and increase in the number of death receptors such as Fas-ligand [8,10]. Another cell surface marker is annexin V, which indicates the expression of phosphatidyl serine on the cell surface. This molecule is found on the inner surface of the cell membrane in normal cells, while in apoptotic cells, it is expressed on the outer surface of the cell membrane. With appropriate DNA staining, this molecule becomes visible, making apoptosis evident in diseased tissue. In our previous study, we demonstrated that annexin V staining, with appropriate preparation techniques in paraffin-embedded tissues, was important in determining apoptosis [9,11]. In this study, annexin V staining was used as a marker of apoptosis in endometriosis, and it was found that it showed a significantly different staining compared to cancer tissue.

Ovarian cancer is an aggressive tumor characterized by a complex immunosuppressive tumor microenvironment, where the immune system’s ability to recognize and destroy tumor cells is impaired. Current cytotoxic immunotherapy options may disrupt this environment, potentially revolutionizing tumor treatment. Studies on apoptosis are important in contributing to this goal [12].

This retrospective study aimed to compare endometriosis cases and ovarian cancer cases in terms of apoptosis and cell proliferation markers’ levels.

## 2. Materials and Methods

For this retrospective study, all pathology specimens from our hospital, a tertiary healthcare institution, were screened. Paraffin blocks from 30 cases of endometrioma and 35 cases of epithelial ovarian cancer were retrospectively evaluated. Approval for this study was obtained from our hospital’s ethics committee (ethics committee approval number 2023/142 dated 12 November 2023).

**Figure 1:**
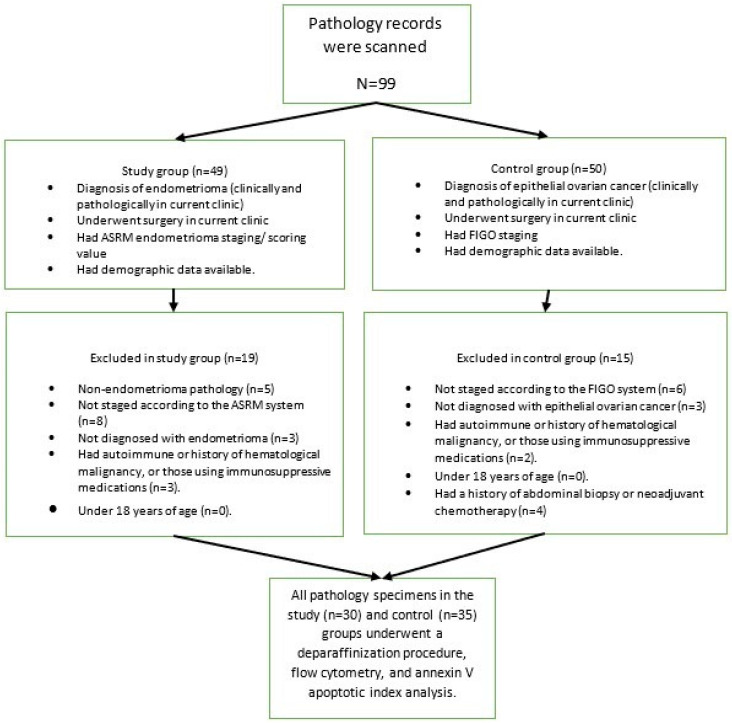
Patient selection flow chart.

For this study, 30 endometrioma patients, who consecutively applied to the clinic where the study was conducted and met the inclusion criteria, were selected as the study group; then, 35 epithelial ovarian cancer patients, who consecutively applied to the clinic where the study was conducted and met the inclusion criteria, were selected as the control group. The flow chart of patient selection is presented in Figure 1.

A study and control group were created. The study group consisted of patients with endometrioma: patients who were diagnosed with endometrioma at the study clinic, underwent surgery, had access to ASRM endometrioma scoring/staging system data [13], had pathological analysis and diagnosis performed at current clinic, and had demographic data available through the hospital’s automated system. Patients with non-endometrioma pathology (benign epithelial ovarian tumor, dermoid, etc.), those not staged according to the ASRM system, those not diagnosed with endometrioma, those with any autoimmune disease, those with a history of hematological malignancy, those using immunosuppressive medications, and those under 18 years of age were excluded from the study.

The control group consisted of patients with a known stage according to the FIGO staging system [14], a pathological diagnosis and evaluation performed at this clinic, surgical treatment at current clinic, a diagnosis of an epithelial ovarian tumor, and clinical/demographic factors available in the hospital automation system records. Patients not having the diagnosis of malign epithelial ovarian tumor, without surgical staging information, those under 18 years of age, with a history of any autoimmune or hematological cancer, or those using immunosuppressive medications were excluded from the study. Patients who had previously received neoadjuvant chemotherapy or had an abdominal biopsy within 6 months were also excluded from the study.

### 2.1. Deparaffinization and Flow Cytometry

Tissues were deparaffinized using the technique detailed in our previous research, and the samples were analyzed using a flow cytometry device. The flow cytometry device also provided information on cell cycle stage ratios, aneuploidy, and annexin V expression rates for apoptosis [9,15,16,17].

To briefly summarize the methodology, paraffin-embedded ovarian tissues were deparaffinized using chemical and mechanical methods. Various DNA stains were then used to determine the cell cycle stage of the cells, the aneuploidy rate, and the apoptotic index using a histogram. Apoptosis was determined using the Annevin V apoptosis kit from BD Pharmingen, catalog number 559763. A BD Accuri C6 Cytometer and its software version 1.0.264.21 were used for flow cytometry analysis.

The device produces a DNA histogram as the result of the analysis. From this histogram, data were obtained that showed the percentage of cells in the G0/G1 phase, G2/M phase, and S phase. The formula (S + G2/M)/(G0/G1 + S + G2/M) × 100 was used to calculate the proliferation index. Annexin V staining curve data were also obtained from another histogram.

### 2.2. Annexin V Apoptotic Index

The basic principle is to measure apoptosis by visualizing phosphatidylserine on the outer surface of apoptotic cells using dyes. In this context, the tissue is treated with a fluorescein-labeled annexin V complex, which makes the expressed phosphatidylserine visible in the ovarian tissue. A flow cytometry device detects these fluorescein-stained cells and provides a staining ratio. The apoptotic index was calculated as follows. The flow cytometry device automatically selects the area with the highest cell density, counts the total number of cells there, and calculates the percentage by dividing the percentage stained with annexin V by the total number of cells (stained and unstained) and multiplying by 100. The resulting percentage was recorded as the apoptotic index [18].

### 2.3. Statistical Analyses

Data were entered into a commercially available statistical program (SPSS version 23.0) by one of the authors, and statistical analysis was performed. Quantitative data were first tested in SPSS for parametric test assumptions (homogeneity and conformity to normal distribution). The Shapiro–Wilk test was used for normal distribution suitability analysis. Then, for quantitative data, comparisons were made between two groups using the Student’s *t* test. For qualitative data, Fisher’s exact chi-square test was used for comparison between each group. The Pearson correlation test was used for correlation analysis of quantitative data, and receiver operating characteristic analysis was performed in SPSS 23 for sensitivity and diagnostic breakpoint analysis. From the Analyze menu, the ROC curve was selected. The annexin apoptotic index value was selected as the test variable, and the ovarian cancer group was selected as the state variable. In the Display section, the ROC curve with the diagonal reference line, standard error and confidence interval, coordinate points of the ROC cure, and the Youden index were selected, and the OK button was pressed. The calculated ROC analysis graph was presented as the sensitivity and specificity. The value with the highest sensitivity and specificity in the coordinate curve table was taken as the cutoff value. A *p* value of <0.05 was accepted as statistically significant.

The sample size and power analysis were calculated using the G*Power 3.1.9.7 computer program. Based on our preliminary study results, the effect size was accepted as 0.86, and, with at least 30 cases in each group, the power was 0.95, and the alpha error rate was 0.05.

## 3. Results

**Figure 2:**
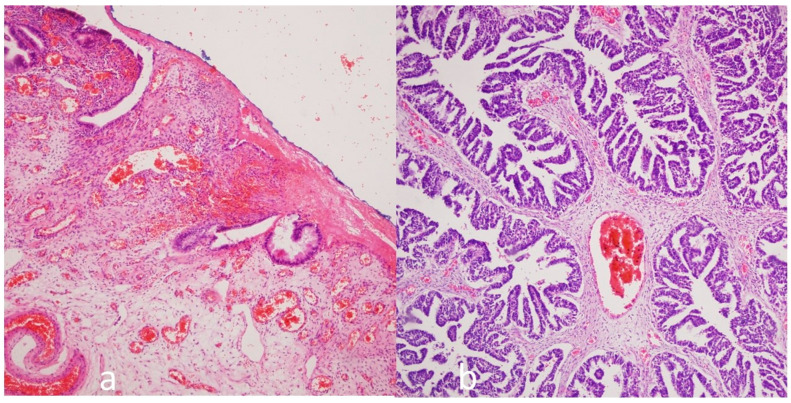
Sample microscopic appearance of ovarian endometrioma (endometrial glands associated with the surface are observed in the endometrioma cyst wall (**a**)) and ovarian carcinoma (neoplastic glands associated with ovarian carcinoma containing papillary structures (**b**)) (Hematoxylin & Eosin staining × 100).

In total, 30 ovarian endometrioma (Figure 2a) and 35 ovarian carcinoma cases (22 were serous ovarian tumors (Figure 2b), 8 were endometroid ovarian tumors, and 3 were mucinous ovarian tumors) were evaluated. A comparison of the demographics of the ovarian endometrioma and carcinoma groups is presented in Table 1. The mean age, parity, and presence of any systemic disease (diabetes mellitus, hypertension, thyroid disease, heart disease, etc.) were found to be statistically significantly higher in the cancer cases. No other statistically significant differences were found between the groups in terms of clinical factors.

A comparison of the G0/G1, G2/M stage, aneuploidy cell ratio, S-phase fraction, proliferative index, and annexin V apoptotic index in ovarian cells in the ovarian endometrioma and carcinoma groups, detected by flow cytometry, is given in Table 2. The G2/M stage cells ratio, S-phase fraction, proliferative index, aneuploidy cell ratio, and annexin V apoptotic index ratio were found to be statistically significantly lower in the endometrioma group compared to the carcinoma group. However, the G0/G1 phase cell ratio was found to be higher in the endometrioma group.

**Figure 3:**
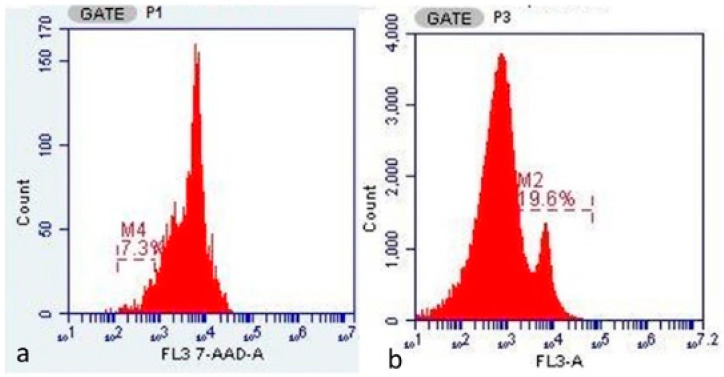
An example of a flow cytometry output showing the aneuploidy cell ratio of one case in the ovarian endometrioma group (**a**) and one case in the ovarian cancer group (**b**). The aneuploidy peak in endometriosis is on the left of the graph (which indicates aneuploidy in favor of hypodiploidy), while the aneuploidy peak in cancer is on the right of the graph (which indicates aneuploidy in favor of hyperdiploidy). 7-AAD (7-Amino-Actinomycin D) is the DNA dye used to assess cell viability in flow cytometry. The abbreviation FL stands for “Fluorescence,” and each number represents a different detector channel. FL3 (Fluorescence Channel 3). The designation 7/AAD-A indicates a measurement made with 7-AAD dye in channel FL3. The letter “A” usually represents the “Area” parameter. In flow cytometry analysis, regions starting with M (Marker) represent analysis regions defined on single-parameter histogram plots. M1, M2, M3, M4: Markers that represent specific fluorescence intensity ranges determined by the investigator on the histogram.

Figure 3a,b show an example of a flow cytometry output with the aneuploidy ratio of one case in the ovarian endometrioma group and one case in the ovarian cancer group. The aneuploidy peak in endometriosis was on the left of the graph (which indicates aneuploidy in favor of hypodiploidy), while the aneuploidy peak in cancer was on the right of the graph (which indicates aneuploidy in favor of hyperdiploidy).

**Figure 4:**
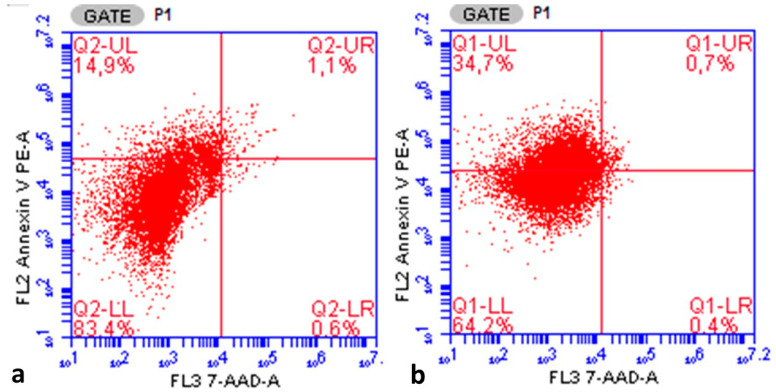
An example of a flow cytometry output showing the annexin V staining ratio of one case in the ovarian endometrioma group (**a**) and one case in the ovarian cancer group (**b**). 7-AAD (7-Amino-Actinomycin D) and PE (phycoerythrin) are the DNA dye used to assess cell viability in flow cytometry. The abbreviation FL stands for “Fluorescence,” and each number represents a different detector channel. FL2 (Fluorescence Channel 2), FL3 (Fluorescence Channel 3). The designation 7/AAD-A indicates a measurement made with 7-AAD dye in channel FL3. The letter “A” usually represents the “Area” parameter. Quadrant Gates. Q1-UL (Quadrant 1—Upper Left), Q2-UR (Quadrant 2—Upper Right), Q1-LL or Q3-LL (Lower Left), Q2-LR or Q4-LR (Lower Right).

**Figure 5:**
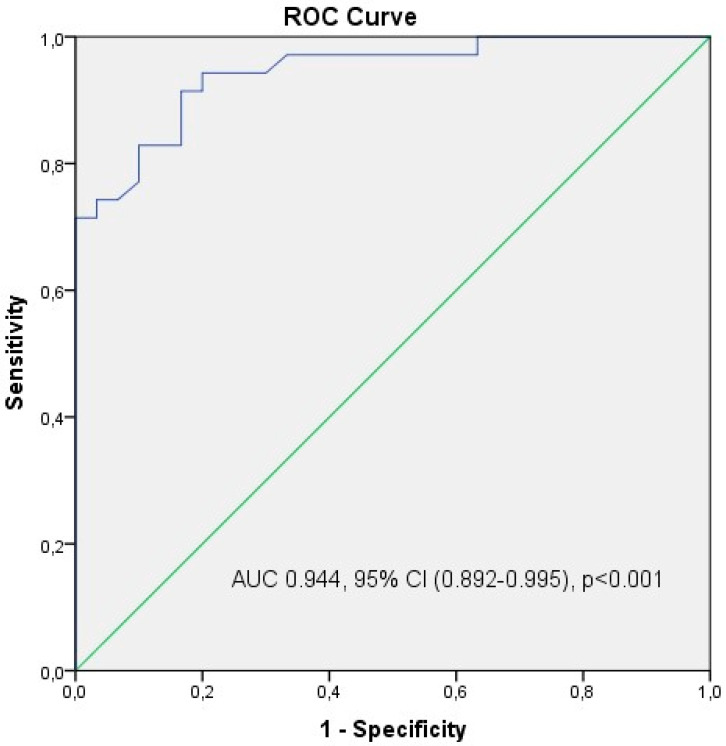
It shows the ROC analysis curve for the annexin V apoptotic index in predicting endometrioma. When the annexin V apoptotic index was below 16.05%, the sensitivity for predicting endometrioma, in other words, in distinguishing it from ovarian cancer, was calculated as 94.3% and the specificity as 80%. AUC; Area under curve, CI; Confidence Interval.

The annexin V apoptotic index was found to be statistically significantly lower in the endometrioma group than in the ovarian cancer group (14.96 ± 7.96% vs. 34.68 ± 7.37%, respectively, *p* < 0.005, Student’s *t* test). Figure 4a,b provide an example of a flow cytometry output showing the annexin V staining ratio of one case in the ovarian endometrioma group and one case in the ovarian cancer group. When evaluated in terms of the entire study group, the annexin V apoptotic index showed statistically significant positive correlations with the S-phase fraction (r = 0.933, *p* < 0.001), aneuploidy cell ratio (r = 0.784, *p* < 0.001), G2/M cell ratio (r = 0.713, *p* < 0.001), and proliferative index (r = 0.923, *p* < 0.001), while it showed a statistically significant negative correlation with the G0/G1 phase cell ratio (r = −0.923, *p* < 0.001). A weak negative correlation was found between age and annexin V expression level in both the endometrioma (r = −0.237, *p* = 0.208) and ovarian cancer groups (r = −0.050, *p* = 0.773). Figure 5 shows the ROC analysis curve for the annexin V apoptotic index in predicting endometrioma (Area under curve 0.944, 95% Confidence Interval 0.892–0.995, *p* < 0.001). When the Annexin V apoptotic index was below 16.05, the sensitivity for endometrioma prediction was calculated as 94.3% and the specificity as 80%. When ROC analysis was performed for G0/G1 phase ratio, G2/M phase ratio, S phase ratio, proliferative index, and aneuploidy cell ratio, no significant diagnostic relevance results were obtained.

## 4. Discussion

In this clinical laboratory-based study, the flow cytometric analysis results and the apoptotic index were compared between patients with endometrioma and ovarian cancer. Flow cytometric analysis determined the cell cycle phases of the ovarian endometrioma and cancer cells included in the analysis. Accordingly, while the proportion of cells in the G0/G1 phase was higher in endometriosis cases compared to the cancer cell population, the proportion of G2/M, S phase, and proliferation index, which are important for proliferation and cell growth, was lower. This supports the notion that endometriosis is a benign disease, with lower proliferative or reproductive tendency than cancer cells. Similarly, when examined in terms of programmed cell death, the apoptotic index of endometriosis cells was also found to be low.

The fundamental questions in our research related to the relationship between endometriosis and cancer. In this context, what is the role of apoptosis? What are the differences between cell cycle changes in endometrioma and ovarian cancer? Can the apoptotic index be used to pathologically distinguish ovarian cancer from endometrioma? This study was designed to answer these questions. Ultimately, we concluded that the apoptotic index and flow cytometry parameters in endometrioma differ from cancer.

It has been previously reported that apoptosis is higher in eutopic endometrial tissue than in ectopic endometrial tissue; in other words, endometriosis develops due to a disruption of the apoptosis mechanism. One study found an apoptosis absorbance value of 0.26 in endometriosis tissue and 0.63 in the normal endometrium [19]. Ectopic endometrial cells likely undergo numerous biochemical and genetic changes to adhere to the ovarian surface. However, they may retain their basic function, and they may form endometriomas, but they also lose the increased apoptotic properties of normal endometrial cells. This makes ectopic endometrial cells resistant to apoptosis. The increase in Bcl-2 and Bax proteins, which are proteins involved in apoptosis, during the late secretory and luteal phases suggest that apoptosis increases during these periods in the normal endometrium, enabling controlled endometrial shedding [20,21]. However, the decrease in apoptosis in endometriosis tissue may suggest that these control mechanisms are lost. In our study, the apoptotic properties, which were significantly increased in cancer tissue, decreased in endometrioma tissue. This situation also supports the literature data.

Flow cytometry is a method that involves collecting individual cells or biological samples in a liquid, arranged in a single row, using a receiver device to measure their physical and chemical properties. Flow cytometry also allows for DNA analysis. It is a rapid and reliable method and can be used with fresh, frozen, formalin-fixed, or paraffin-embedded cell suspensions. DNA changes in the cell population can be detected using fluorescent dyes, and chromosomal aneuploidy can be determined. The stages in the cell’s division cycle can be identified and used in this way [22]. It is now widely used as a diagnostic tool for leukemia, lymphoma, and many solid tumors [23]. According to the flow cytometric analysis results, the endometriosis cases showed DNA activity in terms of cell cycle phases. However, these were not as pronounced as in cancer cells. Similarly, cellular aneuploidy activity was also observed, although less than in cancer. This supports the hypothesis that endometriosis is a precancerous lesion.

Another parameter supporting the precursor role of endometriosis to cancer is presented in Figure 2. The chromosomal diploid structure was disrupted in both cancer and endometriosis. While there was an aneuploidy peak favoring hypodiploidy in endometriosis, there was a peak favoring hyperdiploidy in ovarian cancer. In endometrioma, the potential cellular DNA damage continues, and the irritation from blood residues that were presented inside of endometrioma and the blood product lysis procedure continues. No literature data have been found linking peak hypodiploidy in endometriosis with peak hyperdiploidy in ovarian cancer. However, based on our research findings, our new hypothesis suggests that hypodiploidy in endometriosis may turn into hyperdiploidy in cancer development. However, further large-scale studies with advanced molecular genetics are needed to confirm this finding.

Endometriosis increases the risk of endometroid, clear cell, mucinous, and low- and high-grade serous histological ovarian cancer [7]. Previous studies with small numbers of cases reported an increased risk of clear cell, endometroid, and low-grade serous epithelial ovarian tumors, but not high-grade serous or mucinous tumors [24]. However, newer studies with larger series have reported a significant increase in the risk of all epithelial ovarian cancers (low- and high-grade serous, endometrioid, mucinous, and clear cell tumors) [6].

According to the literature data, endometriosis increases the risk of ovarian cancer by 2–4 times. The presence of ovarian endometriosis carries an additional risk of developing ovarian cancer for every 10,000 women-years of follow-up. All types of epithelial ovarian tumors, not only endometrioid or clear cell ovarian tumors, are increased in the presence of endometriosis [25,26]. Furthermore, the presence of ovarian endometrioma and/or deep infiltrative endometriosis carries the greatest risk. The presence of endometrioma carries an average nine-fold increased risk of ovarian cancer [6]. This requires additional investigation by the pathologist making the endometrioma diagnosis, given the risk of the presence of an ovarian tumor and endometriosis. Therefore, according to our research, the use of flow cytometry annexin V staining is crucial for differential diagnosis. If the annexin V staining level was below 16.05%, a high sensitivity of 94.3% could be used to rule out ovarian cancer. Our detailed literature search did not find any studies on the use of annexin V in differentiating endometriosis and ovarian cancer in this manner. However, the high sensitivity rate may make our research results valuable in this regard.

In our study, the apoptotic index was found to have high sensitivity when used to distinguish cancer from endometriosis. However, its low specificity will pose challenges in clinical use. Elevated CA 125 alone is not sufficient to clinically distinguish between ovarian cancer and endometriosis. However, the specificity increases with the combined use of HE-4 and imaging modalities [27]. As in the results of this study [27], in distinguishing endometriosis from cancer, the addition of biochemical markers, ultrasound findings, magnetic resonance imaging findings, or immunohistochemical markers, in addition to annexin V apoptotic index and flow cytometry, may increase the specificity. However, because our study is the first in the literature and was conducted on paraffin-embedded block specimens, additional testing was not performed. In future studies, with these additional assessments, further tests could increase the specificity to 80%.

One study found coexisting endometriosis in 7.3% of malignant and borderline ovarian tumor cases. Atypical endometriosis was also found in 73% of endometriosis cases [28]. When such cases are presented to the pathologist, they may have difficulty diagnosing cancer. Our research showed that the annexin V apoptotic index has high sensitivity in distinguishing cancer from endometriosis. Even using a specimen embedded in a paraffin block, the pathologist can request flow cytometry analysis, a safe test that is not affected by the paraffin tissue preparation process because it works with cell DNA. This helps the pathologist to diagnose or exclude cancer. This may also prevent unnecessary further surgery and reoperations in cases that do not present with cancer. Likewise, if the pathologist is unsure about the diagnosis of cancer, they can use this test. We believe that flow cytometry testing has a place in the clinical algorithm, especially at these stages.

The underlying cause of ovarian cancer development in endometriosis is unclear. Certain genetic alterations and their continuation can lead to cancer development. The ARID1A gene is expressed in normal endometrium. In endometriosis that develops in the ovary during a process similar to retrograde menstruation, a partial mutation in the ARID1A gene occurs. Over time, a second genomic and epigenomic alteration in this gene can lead to a second mutation, leading to cancer [29]. Similarly, the phosphatidylinositol 3-kinase/protein kinase B/mammalian target of rapamycin pathway, which is related to the ARID1A pathway, may be abnormally activated in endometriosis-associated ovarian cancer. In fact, this genetic pathway may be associated with the balance of cell proliferation, growth, apoptosis, and angiogenesis. However, its aberrant activation may contribute to cancer development [30]. This genetic alteration likely also increases the cell’s apoptosis activity, contributing to the progression to cancer.

Based on our study results, ovarian endometriosis does not always lead to the development of ovarian cancer, and there is insufficient biological evidence to show that abnormal apoptosis is an early marker of ovarian cancer. However, in our study, the lower apoptosis rate in endometriosis compared to ovarian cancer may be helpful in distinguishing ovarian cancer.

No difference was observed in the number of pregnancies between the two groups. However, parity was found to be higher in the ovarian cancer group. Further, both endometriosis [31] and ovarian cancer [32] decreased with increasing parity. We believe that the inclusion of consecutive cases may have contributed to this difference. However, a study on apoptosis in breast epithelium, which is hormone-sensitive like the ovary, reported that apoptosis was not affected by reproductive history [33]. Therefore, it can be concluded that the differences in the reproductive factors in our study did not affect the apoptosis results.

The most significant limitations of our study were the lack of fresh tissue and the small number of cases. Furthermore, the lack of genetic studies, which are other diagnostic parameters of apoptosis, was another limitation. In addition, only annexin V was used as an apoptosis marker. These limitations could be supported by further larger-scale studies.

Another limitation of our study is the statistically significant difference in the mean age of the two groups. However, because endometriosis cases are more common at younger ages, while ovarian cancer cases are more common at older ages, it is highly unlikely that the two groups would be similar in age. According to the correlation analysis results, when the apoptotic index and age correlation were examined separately in both groups, the statistically insignificant negative correlation supported our assertion that the effect of age on the results was limited. However, if possible, future studies with larger series with similar mean ages could clarify this situation.

In our study, the control group had a low number of women with endometroid ovarian tumor histology. This could be considered a weak limiting factor. Although a previous study reported an increased incidence of ovarian tumors in endometrial and clear cell histology and not in serous tumors [24], a new study with a large number of patients [6] reported an increase in all types of ovarian tumors.

## 5. Conclusions

The contributions of our study are as follows: (1) Apoptosis was reduced in endometriosis cases. (2) Cell DNA activity was altered in endometriosis cases, just as in cancer cases. (3) Flow cytometric analysis can be used in the diagnosis of endometriosis in paraffin-embedded tissues. (4) Flow cytometric annexin V analysis provided results in an average of 30 min, making it a promising and highly sensitive differential diagnostic marker that may be used to distinguish between endometriosis and ovarian cancer.

## Figures and Tables

**Table 1:** A comparison of the demographics of the ovarian endometrioma and carcinoma groups.

	Women with Ovarian Endometrioma (n = 30)	Women with Ovarian Carcinoma (n = 35)	*p*
Age ^a^	40.77 ± 6.82	59.63 ± 11.81	<0.001
Parity ^a^	1.37 ± 1.16	2.38 ± 1.89	0.03
Gravida ^a^	1.77 ± 1.52	2.63 ± 2.00	0.11
Systemic disease (%) ^b^			0.005
Absent	12 (40.0%)	8 (25.7%)	
Present	18 (60.0%)	26 (74.3%)	
Hypertension	11 (36.7%)	13(37.4%)	
Diabetes	3 (10.0%)	5 (14.3%)	
Heart disease	-	2 (5.7%)	
Thyroid disease	4 (13.3%)	3 (8.6%)	
Diabetes and hypertension	-	3 (8.6%)	
Surgery ^b^			0.001
Cystectomy	9 (30.0%)	-	
Salpingectomy	6 (20.0%)	5 (14.3%)	
Hysterectomy plus salpingectomy	15 (50.0%)	30 (85.7%)	
International Federation of Gynecology and Obstetrics stage			**-**
Early stage (I–II)	-	14 (40.0%)	
Advanced stage (III–IV)	-	21 (60.0%)	
American Society of Reproductive Medicine stage			**-**
Early stage (I–II)	-	1 (3.3%)	
Advanced stage (III–IV)	-	29 (96.7%)	
Preoperative CA-125 (IU/mL) ^a^	151.67 ± 332.25	264.00 ± 361.55	0.2

^a^ Mean ± standard deviation and ^b^ number of cases (percentages in parentheses). ^b^ Fisher exact chi-square test or ^a^ Student’s *t* test was used for comparison.

**Table 2:** Comparison of the flow cytometric analysis results in the ovarian endometrioma and ovarian carcinoma groups.

	Patients with Endometrioma (n = 30)	Patients with Carcinoma (n = 35)	*p*
G0/G1 phase (%)	90.33 ± 1.29	71.85 ± 4.15	<0.001
G2/M phase (%)	5.66 ± 0.85	9.54 ± 2.08	<0.001
S-phase fraction (%)	4.00 ± 0.65	18.61 ± 3.44	<0.001
Proliferative index (%)	9.67 ± 1.29	28.15 ± 4.15	<0.001
Aneuploidy cell ratio (%)	7.62 ± 1.59	19.70 ± 5.94	<0.001
Annexin V apoptotic index (%)	14.96 ± 7.96	34.68 ± 7.37	<0.001

Student’s *t* test was used for comparison.

## Data Availability

Datasets are available upon request to the corresponding author.

## Abbreviations

The following is a list of abbreviations used in the manuscript:

IGF-1	Insulin-like growth factor-1
IGFBP-3	Insulin-like growth factor binding protein-3
HOMA	Hemostasis model assessment
GnRH	Gonadotrophin-releasing hormone
